# Buffalo Hospital of the Sisters of Charity—Increase of the Medical Stuff, Etc.

**Published:** 1849-12

**Authors:** 


*Buffalo Hospital of the Sisters of Charity—Increase of the Medical Staff, etc.*

The capacity of this Hospital being about to be doubled on the completion of the new building, it has been deemed advisable by the Trustees, with the cordial consent and co-operation of the Faculty of the Medical College, in whom was vested the appointment of the medical and surgical officers of the Institution, to increase the medical staff. For the six months from October 1 to April 1, of each year, the Faculty of the College will retain the medical charge. For the remaining semi-annual period, the following new officers have been appointed:

*Attending Physicians*—E. M. Mackay, M. D., and George N. Burwell, M. D.

*Consulting Physicians*—C. H. Austin, M. D., and Josiah Barnes, M. D.

*Attending Surgeon*—Alden S. Sprague, M. D.

*Consulting Surgeon*—J. E. Camp, M. D.

The other medical officers continue the same as heretofore, viz:

*Attending Surgeon*—Frank H. Hamilton, M. D.

*Consulting Surgeon*—James P. White, M. D.

*Attending Physician*—Austin Flint, M. D.

*Consulting Physician*—Josiah Trowbridge, M. D.

The Directors of the Hospital have offered to the Board of Supervisors to receive sick pauper patients at the average rate of board at the county poor house—60 to 70 cents per week! The Supervisors, at their late meeting, passed a resolution recommending the Superintendents of the Poor to avail themselves of this offer for the benefit of those who elect the hospital instead of the poor house. What course the superintendents will take remains to be seen.

To avoid any occasion for fault finding (in the indulgence of which, toward this Institution, there seems to be a proneness with some,) we are requested to say beforehand, on behalf of the hospital, that incurable cases of disease, or cases in which no benefit is to be expected from medical treatment, will be declined, since the charitable results of the Institution would be materially curtailed by its becoming filled with patients who are not properly subjects for hospital relief. The rejection of cases as unsuitable, will be left to the decision of the medical officers. Patients, also, who have experienced all that is to be expected from hospital treatment, will be liable to be discharged under the advice of the medical officers, to make room for new candidates for relief. The propriety of these rules is too obvious to require remark. They are in conformity with regulations adopted at other similar institutions, and are necessary in order to ensure the greatest amount of good to the greatest number.

